# Trajectories network analysis of chronic diseases among middle-aged and older adults: evidence from the China Health and Retirement Longitudinal Study (CHARLS)

**DOI:** 10.1186/s12889-024-17890-7

**Published:** 2024-02-22

**Authors:** Jiade Chen, Fan Zhang, Yuan Zhang, Ziqiang Lin, Kaisheng Deng, Qingqin Hou, Lixia Li, Yanhui Gao

**Affiliations:** 1 Department of Public Health and Preventive Medicine, School of Medicine, Jinan University, Guangzhou, Guangdong China; 2 Guangdong Provincial Institute of Sports Science, Guangzhou, Guangdong China; 3 School of Public Health, Guangdong Pharmaceutical University, Guangzhou, Guangdong China

**Keywords:** Disease trajectory_1_, Chronic diseases_2_, Middle-aged and older people_3_, China_4_, CHARLS_5_

## Abstract

**Background:**

Given the increased risk of chronic diseases and comorbidity among middle-aged and older adults in China, it is pivotal to identify the disease trajectory of developing chronic multimorbidity and address the temporal correlation among chronic diseases.

**Method:**

The data of 15895 participants from the China Health and Retirement Longitudinal Study (CHARLS 2011 – 2018) were analyzed in the current study. Binomial tests and the conditional logistic regression model were conducted to estimate the associations among 14 chronic diseases, and the disease trajectory network analysis was adopted to visualize the relationships.

**Results:**

The analysis showed that hypertension is the most prevalent disease among the 14 chronic conditions, with the highest cumulative incidence among all chronic diseases. In the disease trajectory network, arthritis was found to be the starting point, and digestive diseases, hypertension, heart diseases, and dyslipidemia were at the center, while memory-related disease (MRD), stroke, and diabetes were at the periphery of the network.

**Conclusions:**

With the chronic disease trajectory network analysis, we found that arthritis was prone to the occurrence and development of various other diseases. In addition, patients of heart diseases/hypertension/digestive disease/dyslipidemia were under higher risk of developing other chronic conditions. For patients with multimorbidity, early prevention can preclude them from developing into poorer conditions, such as stroke, MRD, and diabetes. By identifying the trajectory network of chronic disease, the results provided critical insights for developing early prevention and individualized support services to reduce disease burden and improve patients’ quality of life.

**Supplementary Information:**

The online version contains supplementary material available at 10.1186/s12889-024-17890-7.

## Introduction

In the society of rapid aging, the increased risk of chronic diseases among middle-aged and older adults has become a major public health issue. In China, it was projected that the population of people aged above 60 would reach 402 million (28% of the total population) in 2040, and over 75% are suffered from at least one chronic disease [1, 2], which was referred as “multimorbidity”. Over one third of people with chronic diseases would develop multimorbidity worldwide [3], and the risk increases with age. Multimorbidity can account for 0.15 years of disability (YLDs) [4] and lead to increase cost of medical care [5, 6], and more complicated clinical treatment and medication [7, 8]. Yet, so far, little effective treatment was found for multimorbidity, making it significant to prevent the development of multiple conditions at the early stage. Therefore, it is critical to identify the development trajectory between various chronic conditions.

Despite the high prevalence of multimorbidity, research evidence on the development trajectory pattern between chronic diseases has remained limited. The global prevalence of multimorbidity was 30% among those aged from 45 to 64, and increased to 65% among those aged over 65 [9]. In China, the prevalence of multimorbidity among middle-aged and older adults is similar to the rate abovementioned [10], which is higher than that of India and Brazil [11, 12]. However, most of the existing research on multimorbidity are cross-sectional studies, leaving the trajectory patterns between multiple chronic diseases. Jesen et al. (2014) has conceptualized ‘disease trajectories’ with the temporal correlation between various diseases [13], and developed the data-driven longitudinal analysis has been commonly used to explore disease trajectories in general populations as well as in the clinical samples of specific diseases [14–16]. With a sample of 6.3 million people in Denmark, gout and Chronic obstructive pulmonary disease were identified as key diseases for disease trajectory progression [13]. Patients with septic sweat glands were found to be susceptible to type 1 diabetes and likely to develop acute myocardial infarction, pneumonia and chronic obstructive pulmonary disease subsequently [15]. In the literature on Chinese older population, Meng et al., (2022) reported that cardiovascular and cerebrovascular diseases, cancers, chronic respiratory system diseases and diabetes were the top four chronic diseases with the highest mortality rate in China (i.e., 1615.68, 759.98, 383.27 and 89.44 persons per 100,000 people respectively [17]). However, little has been known about the disease trajectories in Chinese populations, which may be different from those found in European or other populations due to differences in geographical aspects, living habits and genes.

To fill this research gap, the current study has conducted trajectory network analysis with a national cohort data to explore the chronic disease trajectory of people aged 45 and above in China. The research purpose is to unravel the chronic condition trajectory among Chinese mid-life and older adults. The results would advance our understanding about multimorbidity development in middle-aged and older generations, thus providing new insight for potential mechanism or cause and enhancing self-care and disease prevention among people living with chronic conditions.

## Materials and methods

### Study design and data collection

**Fig. 1:**
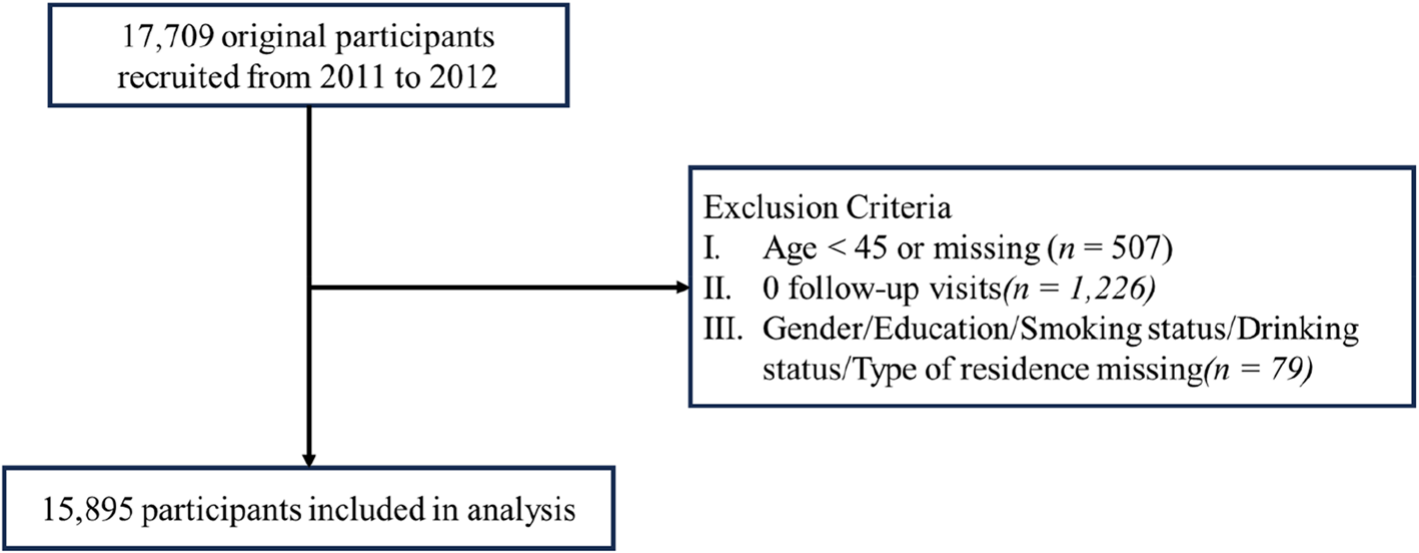
Flow chart through the study

This study used data from the China Health and Retirement Longitudinal Study (CHARLS), a nationally representative sample of people aged 45 and above. The baseline survey was conducted in 2011 with three follow-ups in 2013, 2015, and 2018. With multistage probability sampling, the participants were randomly selected from 450 villages/urban communities in 150 counties/districts of 28 provinces in China. Respondents were interviewed using face-to-face computer-assisted personal interviews. The survey includes demographic characteristics, health status and physical functions, as well as health care and insurance information. Further details about CHARLS are available in the previous publications [18–20]. The current analysis included all the follow-ups since baseline. After excluding the subjects who did not take part in the follow-ups (*n* = 1,226) or have missing values in demographic variables (*n* = 586), 15,895 participants were included in the final analysis (Fig. 1).

### Diseases selection

The diagnosis and onset time of 14 common chronic diseases were collected in baseline and every follow-up through questionnaire. Participants were first asked, "Have you been diagnosed with …(disease) by a doctor?". After receiving a positive answer, the inspector then asked, "When (year or age) was the condition first diagnosed or known by yourself?". Diseases included hypertension, dyslipidemia, diabetes, cancer, chronic lung disease, liver disease, heart diseases, stroke, kidney disease, digestive disease, emotional and mental problems (EMP), memory-related disease (MRD), arthritis, and asthma. In the follow-ups, participants were asked if the last reported disease status was accurate. We corrected for possible recall bias with this question. To capture the trajectory of diseases, the first chronic disease was defined with referring to first chronic disease diagnosed among the 14 diseases. Meanwhile, the age of the incidences of each chronic disease were also reported.

Covariates included gender, age, education, type of residence, smoking, and drinking status. Education includes “low education” (junior high or below) and “high education” (high school or above). The type of residence includes “urban” and “rural” areas. Smoking status included “currently smoking” and “not smoking”. Drinking status included “drinking” (more than once a month) and “not drinking” (less than once a month or None).

### Statistical analysis

SAS 9.4 was used for data analysis. Descriptive results were presented in Table 1. Variables were weighted by individual-level weight with household and non-response adjustment. Box plot was used to describe the median and interquartile range (IQR) of onset age of the first chronic disease. Survival curve was used to describe the development of chronic diseases during the survey. To address the changes in individual’s proportion of chronic disease, stream plot was conducted. Considering the gender differences in the 14 chronic diseases, we conducted all analysis mentioned below (See Supplementary Table 2, Figs S1, S2 and S3). At last, binomial test was employed for assessing the direction of diseases, and conditional logistic regression was conducted to explore the associations between different chronic diseases. All Charts were completed in R 4.2.1.

**Table 1:** Characteristics of population

Variables	Overall (*N* = 15,895)	Variables	Overall (*N* = 15,895)
**Sex, %**		**Cancer, %**	
Male	47.93	No	98.57
Female	52.07	Yes	1.43
**Age (Year)**		**Chronic lung diseases, %**	
≤ 65	74.55	No	85.39
> 65	25.45	Yes	14.61
**Area, %**		**Liver diseases, %**	
Urban	47.06	No	93.85
Rural	52.94	Yes	6.15
**Smoking, %**		**Heart diseases, %**	
Yes	30.43	No	85.19
No	69.57	Yes	14.81
**Drinking, %**		**Stroke, %**	
Yes	33.18	No	95.40
No	66.82	Yes	4.60
**Education, %**		**Kidney diseases, %**	
Middle school below	85.67	No	91.64
High school and above	14.33	Yes	8.36
**Multimorbidity, %**		**Digestive diseases, %**	
0	23.75	No	72.43
1	27.06	Yes	27.57
2	21.49	**EMP, %**	
3	13.38	No	96.80
≥ 4	14.33	Yes	3.20
**Hypertension, %**		**MRD, %**	
No	70.75	No	97.34
Yes	29.25	Yes	2.66
**Dyslipidemia, %**		**Arthritis, %**	
No	86.39	No	59.23
Yes	13.61	Yes	40.77
**Diabetes, %**		**Asthma, %**	
No	92.68	No	94.34
Yes	7.32	Yes	5.66

All variables are weighted by individual level with household and non-response adjustment

### Binomial test

With permutation and combination, there were 91 pairs for 14 chronic diseases. Each pair of diseases had two possible directions of disease progression: disease 1 (D1) → disease 2 (D2) or D2 → D1. Assuming the number of individuals who developed the two diseases consecutively (excluding the number of people who developed two diseases simultaneously) was N, i.e., the number of people developing D1 → D2 was N1 and those with D2 → D1 was N2, and N1 / N and N2 / N follow the binomial distribution of 50%. The sequence of which more people developed two diseases can be regarded as the direction of the disease pair. According to Han et al.(2021) [16], the *p* value threshold was set to 0.05/N (Bonferroni corrected) to reduce type I errors.

### Conditional logistic regression

After the direction was determined for the disease pair by binomial test, e.g., from D1 to D2, D2 would be considered as the outcome and D1 as the exposure of interest. To control for selection bias and test the causal associations, we adopted the method of nested case–control study. Those who had D2 in the follow-up were considered as case group, and matched with control group (who never had D2 and D1 at baseline) by sex, age, and incidence density at a ratio of 1:3. Following this, conditional logistic regression would be conducted to calculate the odds ratio (OR) of D2 after the onset of D1. To minimize bias from small sample sizes, we used the bootstrap for 1000 samples to calculate average ORs and their confidence intervals. [21, 22] and finally selected the disease pairs with a confidence interval not containing 1 and an OR greater than 1 [16].

### Construction of disease trajectory network

The construction of disease trajectory network had previously been described [23]. With connecting the common nodes (i.e., common diseases) between disease pairs to form the disease trajectory, the disease trajectory network was conducted. For example, if D1 → D2 and D2 → D3 were found, then the trajectory of D1 → D2 → D3 would be generated. If D3 → D4 was also found, the trajectory would become D1 → D2 → D3 → D4. Disease trajectory networks are visualized by Cytoscape versions 3.9.1. Each node represents a certain disease, and the color depth of the node represents the number of patients with the disease. The thickness of the line represents the OR value between the two diseases. For details, see in Fig. 6.

### Linear disease trajectory

A linear disease trajectory showed that participants follow three consecutive diseases in the disease trajectory network. The number of participants on each linear trajectory was calculated and used as a sorting criterion. The length between nodes represents the median duration that patients developed from the previous disease to the next one.

## Results

### Characteristics of participants

Table 1 showed the baseline characteristics of the study participants, and among 15,895 participants, 47.93% were male and 52.07% were female. Only 14.33% of participants had obtained high school education or above. Among all participants, 30.43% were smokers and 33.18% were drinkers. The prevalence of multimorbidity (more than two diseases) was 49.20% among the population.

### First-onset age of each chronic disease

**Fig. 2:**
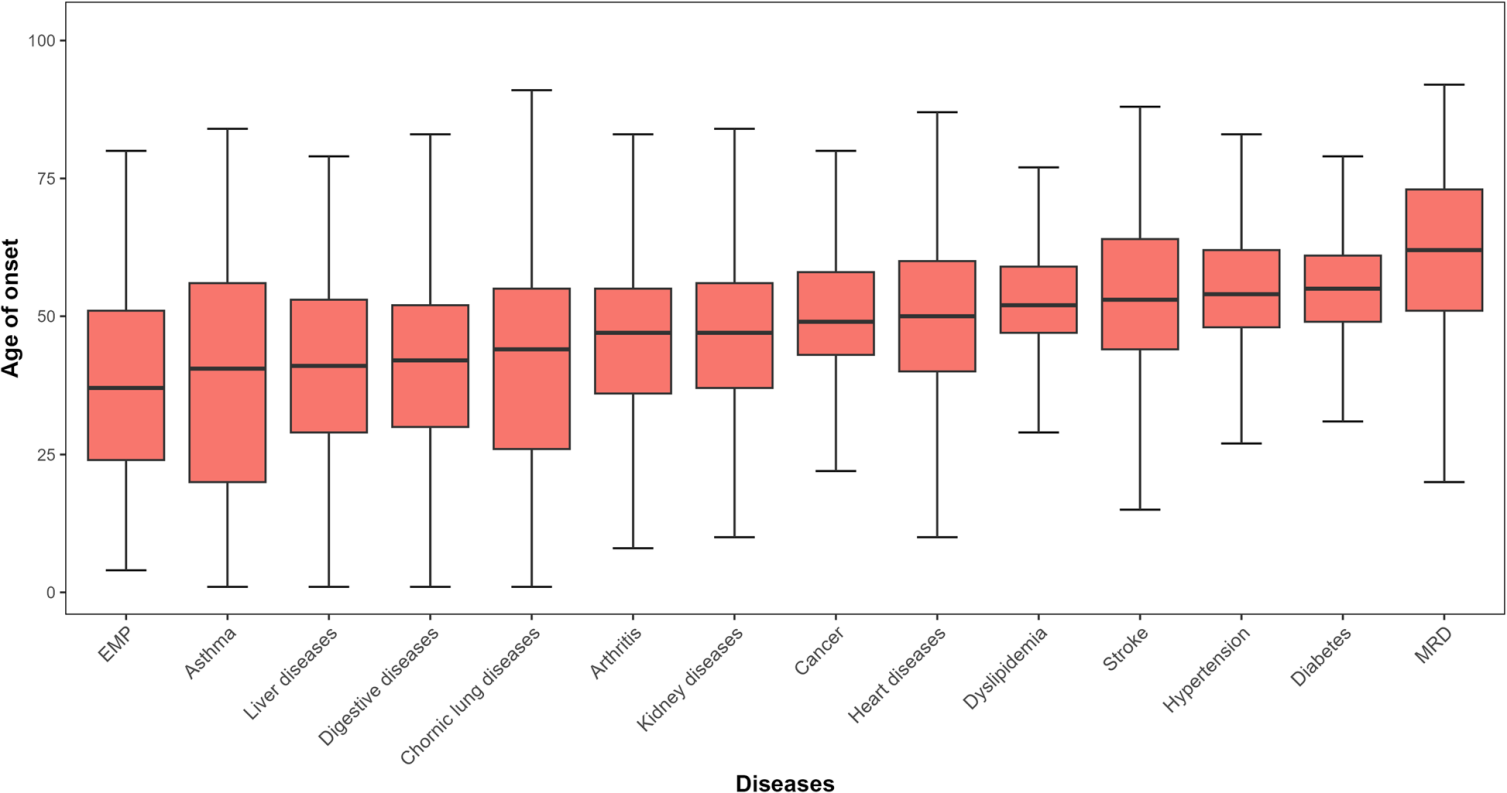
The median and IQR of onset age of individual’s first chronic diseases

Figure 2 showed the median and interquartile range of first-onset age by chronic disease. The onset age of EMP, asthma, liver diseases, and digestive diseases tended to be earlier (all below age 50), while the onset age of stroke, hypertension, diabetes, and MRD were all above 50 years.

### Cumulative occurrence of the chronic diseases

**Fig. 3:**
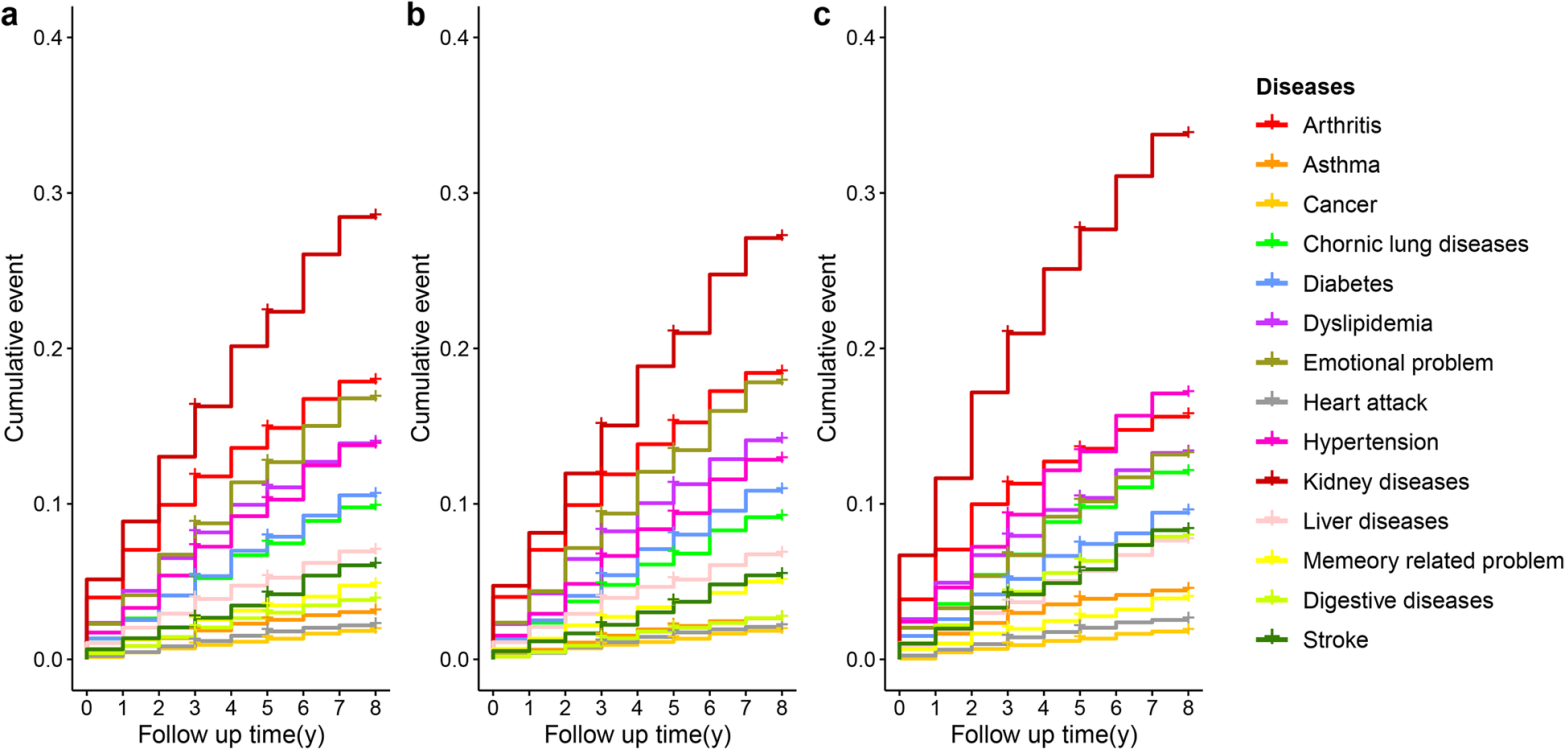
Cumulative rate of 14 chronic diseases

Figure 3 showed the cumulative incidence of 14 chronic diseases. The survival curve was estimated by Kaplan–Meier method. To explore how disease risk varies across different age, two age subgroups were compared. Among overall population,, the cumulative incidence of hypertension, arthritis, and dyslipidemia were the highest in the age group of 45 to 64. Conversely, the cumulative incidence of hypertension, heart diseases, and arthritis were the highest for those aged 65 years and above. Compared with those aged from 45 to 64, the cumulative incidences of all diseases increased among people aged 65 years and above, with the most notable increase in that of heart diseases and hypertension.

### Distributions of diseases across 14 diseases by age and gender

**Fig. 4:**
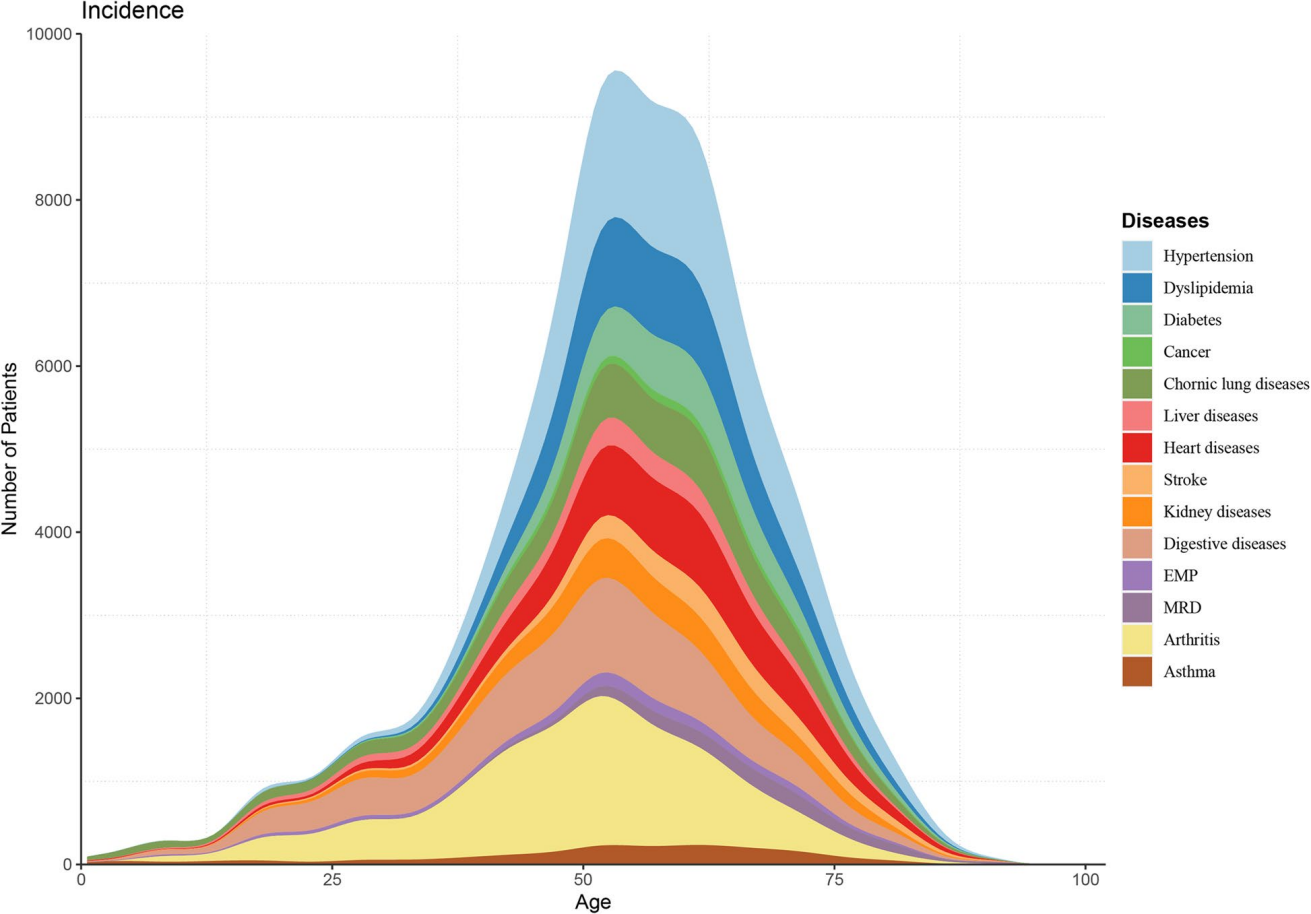
Age distribution of the onset of 14 diseases

Figure 4 displayed how the number of 14 chronic diseases varies across age. The number of diseases began to increase significantly after age 40 except for EMP, asthma, cancer, and MRD, with the most conspicuous raises in arthritis and digestive diseases. Overall, individuals start to develop chronic diseases frequently at the age of 40, reaching a peak of incidence at the age of 53–55.

### The sequence of disease development

**Fig. 5:**
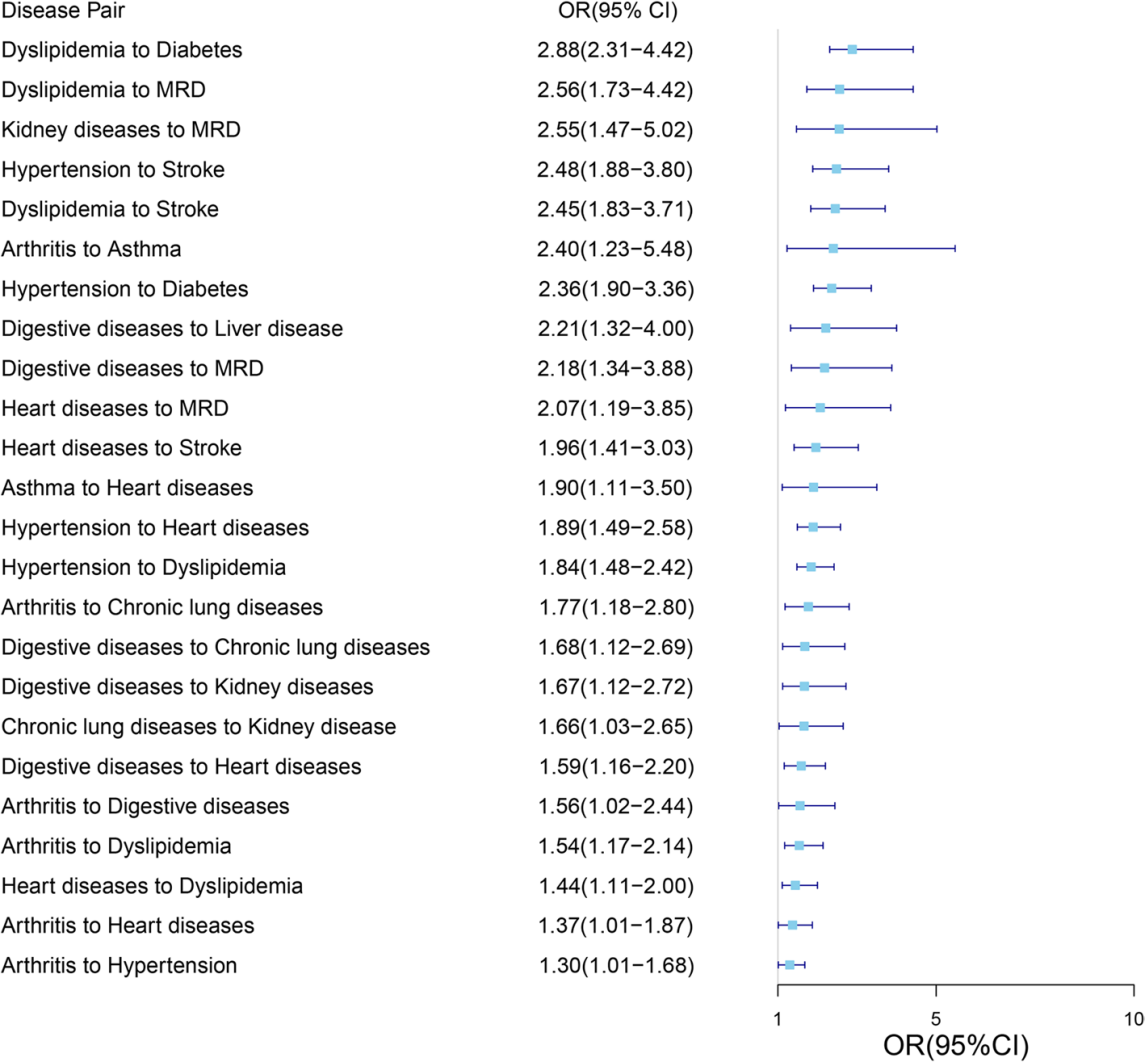
Statistically significant disease pairs

The results of the binomial test were shown in Supplementary Table 1, Additional File 1. In the total sample, 51 pairs of disease were selected, which were presented with descending order of ORs (Fig. 5). The three disease pairs with the highest ORs are: from dyslipidemia to diabetes (OR = 2.88, 95% CI = [2.31–4.42]), from dyslipidemia to MRD (OR = 2.56, 95% CI = [1.73–4.42]), and from kidney diseases to MRD (OR = 2.55, 95% CI = [1.47–5.02]).

### Disease trajectory network

**Fig. 6:**
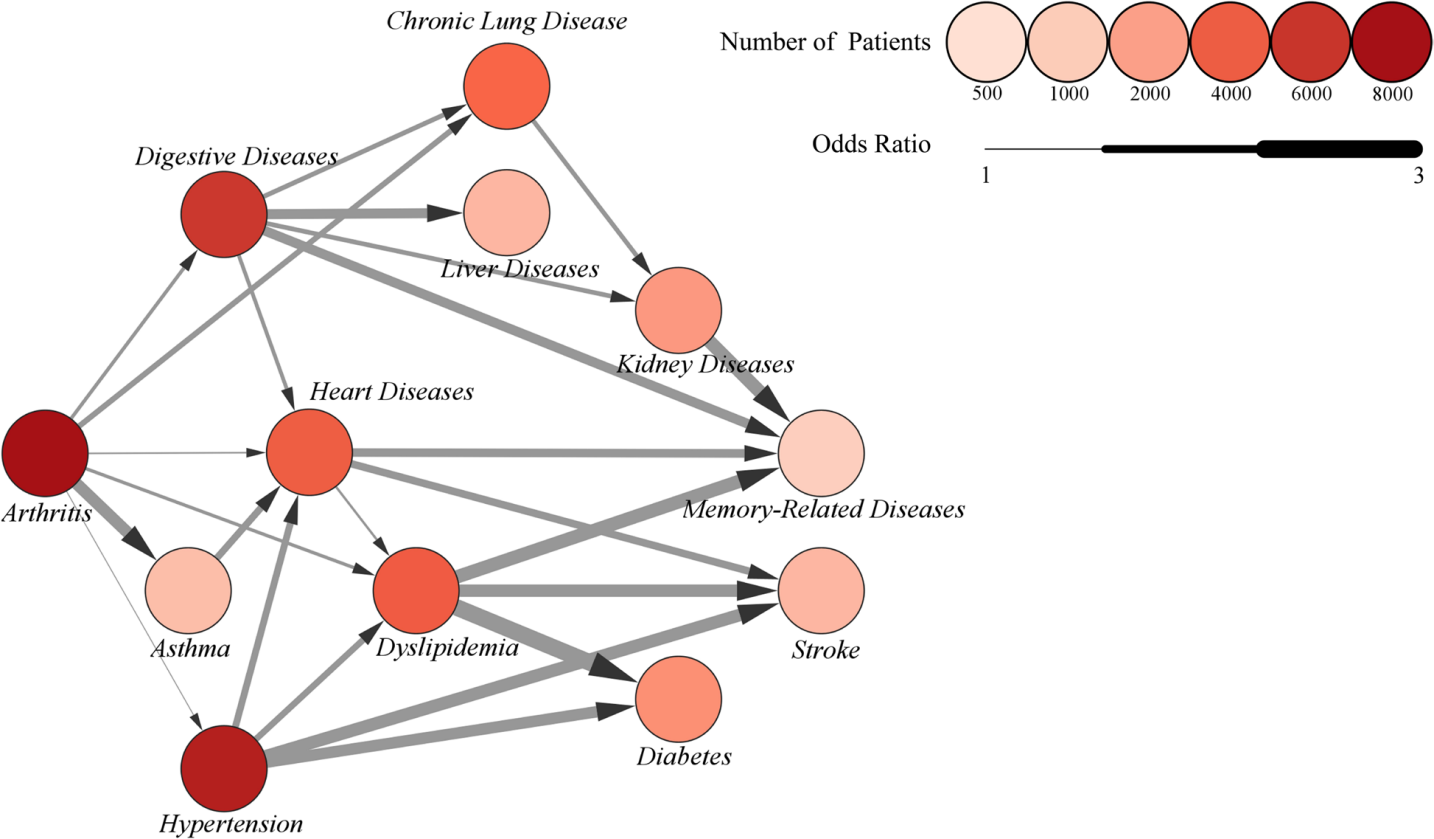
Disease trajectory network. (Nodes represent a certain disease; The color depth of the node represents the number of patients with the disease; The thickness of the line represents the OR value between the two diseases)

Figure 6 showed the trajectory network of chronic diseases. Arthritis was found to be the beginning of the trajectory network. In contrast, stroke and MRD were located at the end of the network. Hypertension, digestive diseases, heart diseases, and dyslipidemia were at the center of the network, indicating that they were associated with or transitional into most other diseases. In addition, there was a trend of increasing risk of disease throughout the disease trajectory network. For instance, the ORs from arthritis, heart diseases, and hypertension developing to dyslipidemia were 1.54 (95% CI: 1.17–2.14), 1.44 (95% CI: 1.11–2.00), and 1.84 (95% CI: 1.48–2.42), respectively. However, the ORs of dyslipidemia developing to stroke, diabetes, and MRD were relatively larger: 2.45 (95% CI: 1.83–3.71), 2.88 (95% CI: 2.31–4.42), and 2.56 (95% CI: 1.73–4.42), respectively. No significant relationship was observed between EMP, cancer, with other diseases.

### Linear trajectory

The trajectories containing three diseases were identified based on the number of patients and the onset age, as shown in Table 2. With the analysis of linear trajectory, it was found that people with arthritis, hypertension and digestive diseases are more likely to develop multimorbidity. The top 10 linear trajectories all start with the three diseases mentioned above. In addition, the average duration from the first stage disease to the intermediate stage disease was 9.5. However, the average duration from the intermediate stage disease to the subsequent disease was 5.1 years, suggesting a more rapid progression of chronic disease in later stage. The top three disease liner trajectories with the largest number of patients are Arthritis-Hypertension-Dyslipidemia (*n* = 446), followed by Arthritis-Hypertension-Heart diseases (*n* = 389) and Arthritis-Hypertension-Diabetes (*n* = 328), which all started from Arthritis.

**Table 2:** Top 10 linear trajectory

Rank	Disease Trajectory			Median age at onset of D1	Number of Patients from D1 to D2	Median time from D1 to D2 (year)	Number of Patients from D1-D2 to D3	Median time from D1-D2 to D3 (year)
	**D1**	**D2**	**D3**					
**1**	Arthritis	Hypertension	Dyslipidemia	44	2693	10	446	4
**2**	Arthritis	Hypertension	Heart diseases	43	2693	11	389	4
**3**	Arthritis	Hypertension	Diabetes	44	2693	10	328	5
**4**	Arthritis	Digestive disease	Heart diseases	40	1869	10	315	7
**5**	Arthritis	Heart diseases	Dyslipidemia	44	1592	10	288	4.5
**6**	Arthritis	Digestive disease	Chronic lung diseases	40	1869	10	275	5
**7**	Digestive disease	Heart diseases	Dyslipidemia	40	1153	10	207	5
**8**	Hypertension	Heart diseases	Dyslipidemia	52	1268	5	203	4
**9**	Arthritis	Hypertension	Stroke	46	2693	10	200	5
**10**	Arthritis	Digestive diseases	Kidney disease	37	1869	9	197	7

## Discussion

With analyzing a large cohort study of middle-aged and older adults in China, we constructed a trajectory network for the progression of chronic diseases. The trajectory network analysis revealed a general pattern of chronic disease development, which filled the gap in the existing research on multimorbidity trajectory in China.

The results indicated that arthritis served as a starting point for developing multimorbidity in this population. The finding is consistent with Castillo et al. (2021), reporting an early onset of arthritis in middle-aged and older populations [24]. According to the authors, not only because arthritis emerged earlier than any other chronic disease, but also because it increased the risk of other diseases. A plausible explanation is that the premature senescence of the immune system in patients with arthritis may lead to multimorbidity [25]. In addition, arthritis was likely to reduce patients’ physical activity, resulting in an elevated risk of diseases. Meanwhile, hypertension, heart diseases, dyslipidemia and digestive diseases were found to be at the center of the trajectory network and involved in various disease courses. This suggests that these four diseases were crucial in the development of chronic diseases among middle-aged and older people. For example, dyslipidemia could be an outcome of cardiovascular disease and was also associated with the onset of metabolic and neurological disorders [26–28]. As for digestive disease, it was found to be related to the polypharmacy in multimorbidity [29, 30]. Furthermore, digestive diseases might affect people’s diets and lifestyle, which increase the risks of other diseases. Heart diseases ensued from a vascular embolism and followed a relatively protracted process. Studies have found that arthritis and hypertension may result in increased risk of heart diseases [31, 32]. Our study also suggested that heart diseases may further lead to stroke, diabetes, and MRD, which was consistent with the previous research [33–38]. Additionally, it was also found that there is a trend of increasing risk throughout the disease trajectory network. These findings would provide critical insights for the time window of designing disease prevention services.

By addressing linear trajectory, it was found that the progression of multimorbidity was relatively slow at the beginning compared to the later stage. After the onset of the first chronic disease, it took more years for the subsequent disease to occur. Once the second disease emerges, the progression into other diseases tended to be faster. Therefore, more attention should be paid to preventing future disease development, particularly after the onset of the first chronic condition. Once it started, developing into multimorbidity would be faster and more difficult to prevent. In other words, prevention-focused strategies are more important and beneficial than treatment-focused strategies in controlling for chronic diseases development. The findings help clarify the prevention priorities for multimorbidity in middle-aged and older people in China, and has practical implications for developing individualized prevention strategies to reduce the medical burden and improve the quality of life among patients with chronic conditions.

Our study is based on nationally representative cohort, representing the Chinese middle-aged and older people. Besides, temporal disease trajectory analysis is used to visualize pattern of chronic condition progression. Most importantly, our results can provide insights for the time window and the focus of chronic disease control service. There are also some limitations to be acknowledged. First, the small sample size in our study may limit the generalizability of the results to the wider population. We acknowledge that a larger population would allow for more statistical power. However, we have employed rigorous statistical analysis to ensure the validity of our results. In future research endeavors, sample size will be a primary consideration for us. Second, the prevalence of chronic diseases may have been affected by participants’ recall bias given the nature of self-report data. Future studies should consider including medical record into the analysis. Third, the entire follow-up periods have lasted for only seven years, which did not capture the occurrence of diseases with slow onset and complex causes. In addition, since only 14 common chronic diseases were included in the survey it is difficult to compare the results of studies from other countries. More chronic conditions should be included in future studies. At last, the classifications of diseases were broad. The aggregation of diverse diseases into 14 categories may obscure the nuanced understanding. It is important for future research to consider more granular disease classifications.

## Conclusion

With the disease trajectory network analysis, we found that arthritis was the key disease that was prone to the occurrence and development of various other diseases. In addition, patients with heart diseases/ hypertension/ digestive disease/ dyslipidemia were under higher risk of developing other chronic conditions. The results highlighted that for patients with multimorbidity, early prevention can reduce the risks of developing chronic diseases with a poorer prognosis, such as stroke, MRD, and diabetes. By identifying the trajectory network of chronic disease, the results provided critical insights for developing early prevention and individualized service to better control the progression of multimorbidity among middle-aged and older adults, as well as reduce disease burden and improve their quality of life.

### Supplementary Information

**Additional file 1: Supplementary Table 1.** Results of the binomial test.** Supplementary Table 2.** Characteristics of Population.** Fig S1.** The median and IQR of onset age of individual’s first chronic diseases by gender.** Fig S2.** Cumulative rate of 14 chronic diseases.** Fig S3.** Age distribution of the onset of 14 diseases by gender.

## Data Availability

The dataset that support the findings of this study are available on the Charls website (http://charls.pku.edu.cn/en/) [39].

## Abbreviations

CHARLS: China Health and Retirement Longitudinal Study
EMP: Emotional and Mental Problems
MRD: Memory-Related Disease

## References

[1] Ageing and health in China 2023. https://www.who.int/china/health-topics/ageing. Accessed 25 Jul 2023.

[2] National Health Commission of the People’s Republic of China Healthy China action. http://www.nhc.gov.cn/guihuaxxs/s3585u/201907/e9275fb95d5b4295be8308415d4cd1b2.shtml. Accessed 25 Jul 2023.

[3] Nguyen H, Manolova G, Daskalopoulou C, Vitoratou S, Prince M, Prina AM (2019). Prevalence of multimorbidity in community settings: A systematic review and meta-analysis of observational studies. J Comorb.

[4] Zhou X, Zhang D, Health P (2021). Multimorbidity in the elderly: a systematic bibliometric analysis of research output. Int J Environ Res Public Health.

[5] Soley-Bori M, Ashworth M, Bisquera A, Dodhia H, Lynch R, Wang Y (2021). Impact of multimorbidity on healthcare costs and utilisation: a systematic review of the UK literature. Bri J Gen Pract.

[6] Noël PH, Chris Frueh B, Larme AC, Pugh JA (2005). Collaborative care needs and preferences of primary care patients with multimorbidity. Health Expect.

[7] Schneider J, Algharably EAE, Budnick A, Wenzel A, Dräger D, Kreutz RJ. High prevalence of multimorbidity and polypharmacy in elderly patients with chronic pain receiving home care are associated with multiple medication-related problems. 2021:1180. 10.3389/fphar.2021.686990 PMC8217758 34168565

[8] Molist-Brunet N, Sevilla-Sánchez D, Puigoriol-Juvanteny E, Bajo-Peñas L, Cantizano-Baldo I, Cabanas-Collell L (2022). Individualized medication review in older people with multimorbidity: a comparative analysis between patients living at home and in a nursing home. Int J Environ Res Public Health.

[9] Skou ST, Mair FS, Fortin M, Guthrie B, Nunes BP, Miranda JJ (2022). Multimorbidity. Nat Rev Dis Primers.

[10] Su Z, Huang L, Zhu J, Cui S. Effects of multimorbidity coexistence on the risk of mortality in the older adult population in China. Front Public Health. 2023;11:1110876. 10.3389/fpubh.2023.1110876. 10.3389/fpubh.2023.1110876 PMC10113675 37089511

[11] Hone T, Stokes J, Trajman A, Saraceni V, Coeli CM, Rasella D (2021). Racial and socioeconomic disparities in multimorbidity and associated healthcare utilisation and outcomes in Brazil: a cross-sectional analysis of three million individuals. BMC Public Health.

[12] Boro B, Saikia N (2022). Association of multimorbidity and physical activity among older adults in India: an analysis from the longitudinal ageing survey of India (2017–2018). BMJ Open.

[13] Jensen AB, Moseley PL, Oprea TI, Ellesøe SG, Eriksson R, Schmock H (2014). Temporal disease trajectories condensed from population-wide registry data covering 6.2 million patients. Nat Commun.

[14] Yang H, Pawitan Y, He W, Eriksson L, Holowko N, Hall P (2019). Disease trajectories and mortality among women diagnosed with breast cancer. Breast Cancer Res.

[15] Andersen RK, Jørgensen IF, Reguant R, Jemec GBE, Brunak S (2020). Disease trajectories for hidradenitis suppurativa in the Danish population. JAMA Dermatol.

[16] Han X, Hou C, Yang H, Chen W, Ying Z, Hu Y (2021). Disease trajectories and mortality among individuals diagnosed with depression: a community-based cohort study in UK Biobank. Mol Psychiatry.

[17] Meng S-D, Wang W, Ying P, Wang L-J, Liu Y-N, Liu J-M (2022). Analysis on the burden of four major chronic diseases among the elderly (≥60 years old) in China in 2005 and 2020. Chin J Prev Contr Chronic Dis.

[18] Zhao Y, Hu Y, Smith JP, Strauss J, Yang G (2014). Cohort profile: the China health and retirement longitudinal study (CHARLS). Int J Epidemiol.

[19] Liu Y. China health and retirement longitudinal study wave 4 user’s guide.

[20] Zhao Y, Strauss J, Yang G, Giles J, Hu P, Hu Y, et al. China health and retirement longitudinal study-2011-2012 national baseline users’ guide. Beijing: National School of Development, Peking University; 2013. p. 1–56. https://charls.pku.edu.cn/en/doc/User2011.pdf.

[21] Moisset X, Perié M, Pereira B, Dumont E, Lebrun-Frenay C, Lesage F-X (2017). Decreased prevalence of cancer in patients with multiple sclerosis: a case-control study. Plos One.

[22] Adjei IA, Karim R (2016). An application of bootstrapping in logistic regression model. Open Access Lib J.

[23] Siggaard T, Reguant R, Jørgensen IF, Haue AD, Lademann M, Aguayo-Orozco A (2020). Disease trajectory browser for exploring temporal, population-wide disease progression patterns in 7.2 million Danish patients. Nat Commun.

[24] Castillo-Cañón JC, Trujillo-Cáceres SJ, Bautista-Molano W, Valbuena-García AM, Fernández-Ávila DG, Acuña-Merchán L (2021). Rheumatoid arthritis in Colombia: a clinical profile and prevalence from a national registry. Clin Rheumatol.

[25] Lindstrom TM, Robinson WH (2010). Rheumatoid arthritis: a role for immunosenescence?. J Am Geriatr Soc.

[26] Krist AH, Davidson KW, Mangione CM, Barry MJ, Cabana M, Caughey AB (2020). Behavioral counseling interventions to promote a healthy diet and physical activity for cardiovascular disease prevention in adults with cardiovascular risk factors: US Preventive Services Task Force recommendation statement. JAMA.

[27] Abbasi A, Corpeleijn E, Gansevoort RT, Gans RO, Hillege HL, Stolk RP (2013). Role of HDL cholesterol and estimates of HDL particle composition in future development of type 2 diabetes in the general population: the PREVEND study. J Clin Endocrinol Metab.

[28] Reitz C (2013). Dyslipidemia and the risk of Alzheimer’s disease. Curr Atheroscler Rep.

[29] Herzig K-H, Purhonen A-K, Räsänen KM, Idziak J, Juvonen P, Phillps R (2011). Fecal pancreatic elastase-1 levels in older individuals without known gastrointestinal diseases or diabetes mellitus. BMC Geriatr.

[30] Du Y, Su T, Song X, Gao J, Zou D, Zuo C (2014). Efficacy and safety of cinitapride in the treatment of mild to moderate postprandial distress syndrome–predominant functional dyspepsia. J Clin Gastroenterol.

[31] Semb AG, Ikdahl E, Wibetoe G, Crowson C, Rollefstad S (2020). Atherosclerotic cardiovascular disease prevention in rheumatoid arthritis. Nat Rev Rheumatol.

[32] Han M, Chen Q, Liu L, Li Q, Ren Y, Zhao Y (2020). Stage 1 hypertension by the 2017 American College of Cardiology/American Heart Association hypertension guidelines and risk of cardiovascular disease events: systematic review, meta-analysis, and estimation of population etiologic fraction of prospective cohort studies. J Hypertens.

[33] Chen H, Zhou Y, Huang L, Xu X, Yuan C (2024). Multimorbidity burden and developmental trajectory in relation to later-life dementia: a prospective study. Alzheimers Dement..

[34] Demant MN, Gislason GH, Køber L, Vaag A, Torp-Pedersen C, Andersson CJD (2014). Association of heart failure severity with risk of diabetes: a Danish nationwide cohort study. Diabetologia.

[35] Islam MR, Lbik D, Sakib MS, Maximilian Hofmann R, Berulava T, Jiménez Mausbach M (2021). Epigenetic gene expression links heart failure to memory impairment. EMBO Mol Med.

[36] Aradine E, Hou Y, Cronin CA, Chaturvedi S (2020). Chaturvedi S, Current status of dyslipidemia treatment for stroke prevention. Current neurology and neuroscience reports.

[37] Vergès B (2020). Metabolism. Dyslipidemia in type 1 diabetes: a masked danger. Trends Endocrinol Metab.

[38] Silva MVF (2019). Loures CdMG, Alves LCV, de Souza LC, Borges KBG, Carvalho MdGJJobs. Alzheimer’s disease: risk factors and potentially protective measures.

[39] China Health and Retirement Longitudinal Survey. http://charls.pku.edu.cn/en/. Accessed 25 Jul 2023.

