# Supplementary material for: Trajectories network analysis of chronic diseases among middle-aged and older adults: evidence from the China Health and Retirement Longitudinal Study (CHARLS)
Source: BMC Public Health. 2024 Feb 22;24:559. doi: 10.1186/s12889-024-17890-7 (PMC10882875; doi:10.1186/s12889-024-17890-7)
Supplement: Supplementary file 1 — Additional file 1: Supplementary Table 1. Results of the binomial test. Supplementary Table 2. Characteristics of Population. Fig S1. The median and IQR of onset age of individual’s first chronic diseases by gender. Fig S2. Cumulative rate of 14 chronic diseases. Fig S3. Age distribution of the onset of 14 diseases by gender. [file 12889_2024_17890_MOESM1_ESM.docx]

**Supplementary Table 1 Results of the binomial test**

| Disease 1 | Disease 2 | D1→D2 | D2→D1 | *P* |
| --- | --- | --- | --- | --- |
| Hypertension | Dyslipidemia | 1378 | 587 | 0.0001 |
| Hypertension | Diabetes | 1012 | 331 | 0.0001 |
| Hypertension | Cancer | 136 | 72 | 0.0001 |
| Hypertension | Chronic lung diseases | 639 | 957 | 0.0001 |
| Hypertension | Heart diseases | 1268 | 824 | 0.0001 |
| Hypertension | Stroke | 660 | 208 | 0.0001 |
| Hypertension | Digestive diseases | 802 | 1650 | 0.0001 |
| Hypertension | MRD | 391 | 113 | 0.0001 |
| Hypertension | Arthritis | 912 | 2693 | 0.0001 |
| Hypertension | Asthma | 236 | 423 | 0.0001 |
| Dyslipidemia | Diabetes | 529 | 350 | 0.0001 |
| Dyslipidemia | Chronic lung diseases | 298 | 621 | 0.0001 |
| Dyslipidemia | Heart diseases | 603 | 801 | 0.0001 |
| Dyslipidemia | Stroke | 304 | 210 | 0.0001 |
| Dyslipidemia | Kidney diseases | 311 | 397 | 0.0012 |
| Dyslipidemia | Digestive diseases | 397 | 1187 | 0.0001 |
| Dyslipidemia | MRD | 203 | 109 | 0.0001 |
| Dyslipidemia | Arthritis | 405 | 1628 | 0.0001 |
| Dyslipidemia | Asthma | 110 | 289 | 0.0001 |
| Diabetes | Chronic lung diseases | 165 | 388 | 0.0001 |
| Diabetes | Liver disease | 127 | 181 | 0.0021 |
| Diabetes | Heart diseases | 326 | 497 | 0.0001 |
| Diabetes | Kidney diseases | 195 | 268 | 0.0007 |
| Diabetes | Digestive diseases | 203 | 688 | 0.0001 |
| Diabetes | EMP | 48 | 103 | 0.0001 |
| Diabetes | Arthritis | 217 | 1037 | 0.0001 |
| Diabetes | Asthma | 56 | 177 | 0.0001 |
| Cancer | Digestive diseases | 64 | 125 | 0.0001 |
| Cancer | Arthritis | 62 | 179 | 0.0001 |
| Chronic lung diseases | Liver disease | 279 | 207 | 0.0011 |
| Chronic lung diseases | Heart diseases | 658 | 458 | 0.0001 |
| Chronic lung diseases | Stroke | 238 | 110 | 0.0001 |
| Chronic lung diseases | Kidney diseases | 368 | 290 | 0.0024 |
| Chronic lung diseases | Digestive diseases | 624 | 928 | 0.0001 |
| Chronic lung diseases | MRD | 182 | 80 | 0.0001 |
| Chronic lung diseases | Arthritis | 750 | 1329 | 0.0001 |
| Liver disease | Digestive diseases | 262 | 507 | 0.0001 |
| Liver disease | EMP | 38 | 75 | 0.0005 |
| Liver disease | Arthritis | 271 | 604 | 0.0001 |
| Liver disease | Asthma | 65 | 112 | 0.0004 |
| Heart diseases | Stroke | 327 | 186 | 0.0001 |
| Heart diseases | Digestive diseases | 575 | 1152 | 0.0001 |

**(Continued** **Supplementary Table 1)**

| Heart diseases | MRD | 241 | 105 | 0.0001 |
| --- | --- | --- | --- | --- |
| Heart diseases | Arthritis | 577 | 1592 | 0.0001 |
| Heart diseases | Asthma | 185 | 327 | 0.0001 |
| Stroke | Digestive diseases | 131 | 378 | 0.0001 |
| Stroke | Arthritis | 161 | 617 | 0.0001 |
| Stroke | Asthma | 45 | 107 | 0.0001 |
| Kidney diseases | Digestive diseases | 341 | 664 | 0.0001 |
| Kidney diseases | MRD | 131 | 70 | 0.0001 |
| Kidney diseases | Arthritis | 387 | 944 | 0.0001 |
| Kidney diseases | Asthma | 100 | 160 | 0.0002 |
| Digestive diseases | EMP | 238 | 145 | 0.0001 |
| Digestive diseases | MRD | 295 | 65 | 0.0001 |
| Digestive diseases | Arthritis | 1578 | 1869 | 0.0001 |
| EMP | Arthritis | 158 | 334 | 0.0001 |
| MRD | Arthritis | 73 | 429 | 0.0001 |
| MRD | Asthma | 32 | 94 | 0.0001 |
| Arthritis | Asthma | 460 | 313 | 0.0001 |

Gender specific disease descriptions:

| **Supplementary Table 2 Characteristics of Population** | | | |  |
| --- | --- | --- | --- | --- |
| **Variables** | **Male (N=7,681)** | **Female (N=8,214)** | **P Value** | |
| **Age (Year)** |  |  | <.0001 | |
| Median (IQR) | 58 (52–66) | 51 (58–66) |  | |
| **Area, %** |  |  | <.0001 | |
| Urban | 46.12 | 47.92 |  | |
| Rural | 53.88 | 52.08 |  | |
| **Smoking, %** |  |  | <.0001 | |
| Yes | 56.90 | 6.07 |  | |
| No | 43.10 | 93.93 |  | |
| **Drinking, %** |  |  | <.0001 | |
| Yes | 56.46 | 11.75 |  | |
| No | 43.54 | 88.25 |  | |
| **Education, %** |  |  | <.0001 | |
| Middle school and below | 81.14 | 89.84 |  | |
| High school and above | 18.86 | 10.16 |  | |
| **Multimorbidity, %** |  |  | <.0001 | |
| 0 | 25.21 | 22.40 |  | |
| 1 | 27.74 | 26.43 |  | |
| 2 | 21.07 | 21.87 |  | |
| 3 | 12.78 | 13.93 |  | |
| >4 | 13.19 | 15.37 |  | |

**(Continued Supplementary Table 2)**

| **Hypertension, %** |  |  | <.0001 |
| --- | --- | --- | --- |
| No | 71.92 | 69.68 |  |
| Yes | 28.08 | 30.32 |  |
| **Dyslipidemia, %** |  |  | <0.001 |
| No | 86.50 | 86.28 |  |
| Yes | 13.50 | 13.72 |  |
| **Diabetes, %** |  |  | <.0001 |
| No | 92.90 | 92.48 |  |
| Yes | 7.10 | 7.52 |  |
| **Cancer, %** |  |  | <.0001 |
| No | 99.04 | 98.14 |  |
| Yes | 0.96 | 1.86 |  |
| **Chronic lung diseases, %** |  |  | <.0001 |
| No | 83.31 | 87.30 |  |
| Yes | 6.48 | 5.84 |  |
| **Liver diseases, %** |  |  | <.0001 |
| No | 93.52 | 94.16 |  |
| Yes | 6.48 | 5.84 |  |
| **Heart diseases, %** |  |  | <.0001 |
| No | 88.21 | 82.40 |  |
| Yes | 11.79 | 17.60 |  |
| **Stroke, %** |  |  | <.0001 |
| No | 95.04 | 95.74 |  |
| Yes | 4.96 | 4.26 |  |
| **Kidney diseases, %** |  |  | <.0001 |
| No | 91.04 | 92.20 |  |
| Yes | 8.96 | 7.80 |  |
| **Digestive diseases, %** |  |  | <.0001 |
| No | 74.51 | 70.52 |  |
| Yes | 25.49 | 29.48 |  |
| **EMP, %** |  |  | <.0001 |
| No | 97.71 | 95.96 |  |
| Yes | 2.29 | 4.04 |  |
| **MRD, %** |  |  | <.0001 |
| No | 97.18 | 97.50 |  |
| Yes | 2.82 | 2.50 |  |
| **Arthritis, %** |  |  | <.0001 |
| No | 63.72 | 55.11 |  |
| Yes | 36.28 | 44.89 |  |
| **Asthma, %** |  |  | <.0001 |
| No | 93.37 | 95.23 |  |
| Yes | 6.63 | 4.77 |  |
| All variables are weighted by individual level with household and non-response adjustment. | | | |

Wilcox test was used to compare whether the difference in age of onset of the same disease across gender was significant. The symbol * indicates statistically significance.


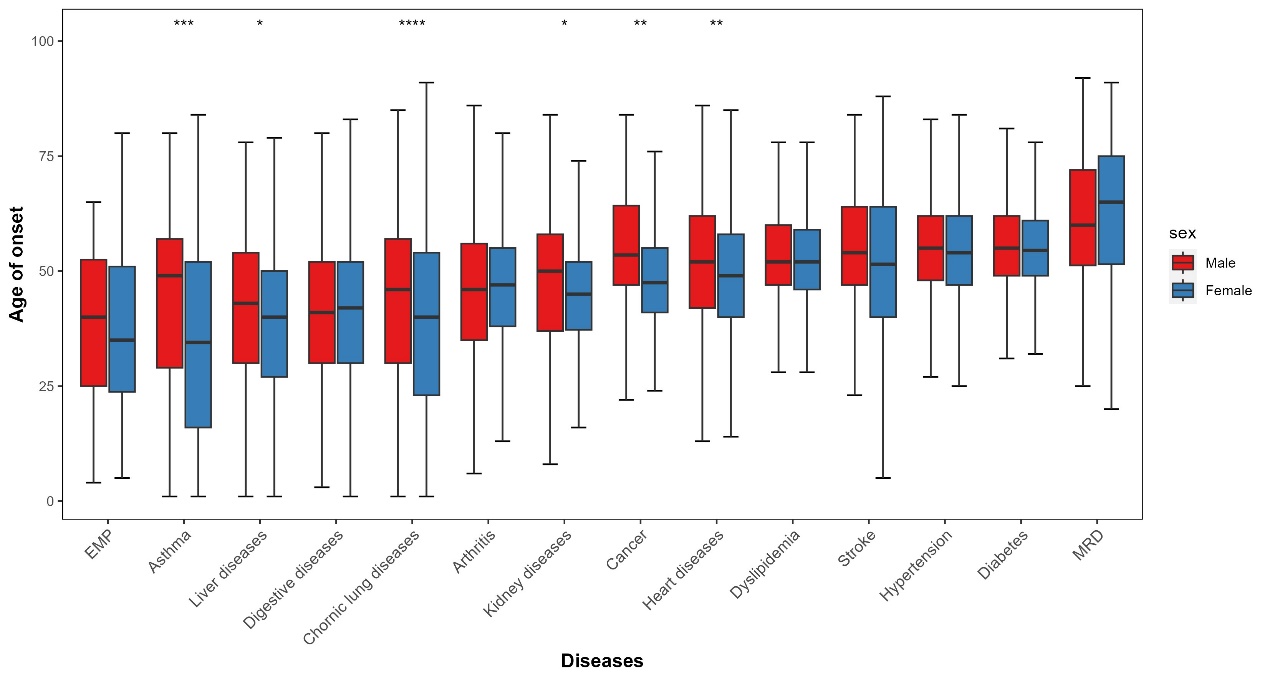


**Fig** **S1. The median and IQR of onset age of individual’s first chronic diseases by gender**


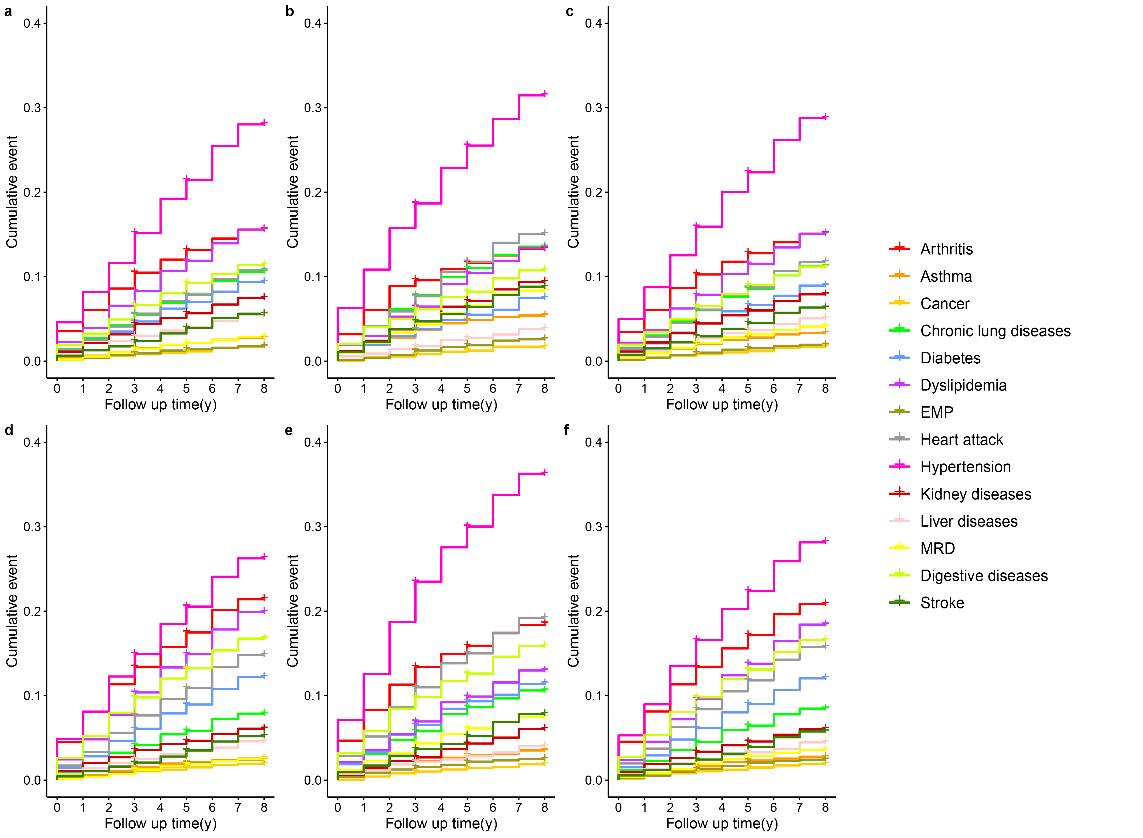


**Fig S2. Cumulative rate of 14 chronic diseases**

((a), (b), (c) is for male and (d), (e), (f) is for female,

(a), (d) is for people aged 45-65, (b), (e) is for people aged 65 and above, (c), (f) is for the all-age group)


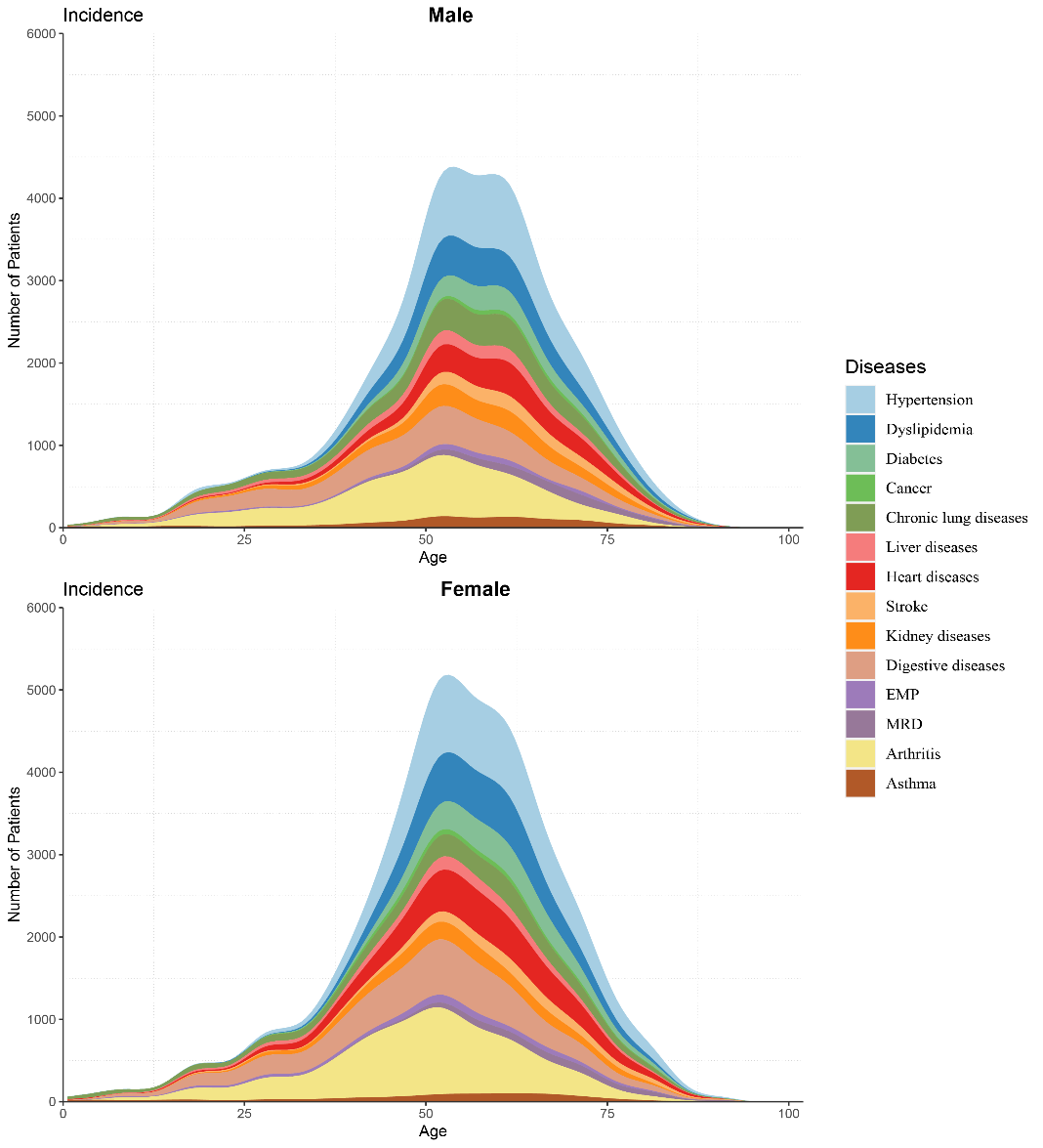


**Fig S3. Age distribution of the onset of 14 diseases by gender**
